# In-situ electron microscopy mapping of an order-disorder transition in a superionic conductor

**DOI:** 10.1038/s41467-019-09502-5

**Published:** 2019-04-03

**Authors:** Jaeyoung Heo, Daniel Dumett Torres, Progna Banerjee, Prashant K. Jain

**Affiliations:** 1 Department of Materials Science & Engineering, University of Illinois at Urbana−Champaign, Urbana, IL 61801 USA; 2 Department of Chemistry, University of Illinois at Urbana−Champaign, Urbana, IL 61801 USA; 3 Department of Physics, University of Illinois at Urbana−Champaign, Urbana, IL 61801 USA; 4 Materials Research Laboratory, University of Illinois at Urbana−Champaign, Urbana, IL 61801 USA; 5 Beckman Institute of Advanced Science and Technology, University of Illinois at Urbana−Champaign, Urbana, IL 61801 USA

**Keywords:** Imaging studies, Materials for energy and catalysis, Nanoparticles

## Abstract

Solid-solid phase transitions are processes ripe for the discovery of correlated atomic motion in crystals. Here, we monitor an order-disorder transition in real-time in nanoparticles of the super-ionic solid, Cu_2−x_Se. The use of in-situ high-resolution transmission electron microscopy allows the spatiotemporal evolution of the phase transition within a single nanoparticle to be monitored at the atomic level. The high spatial resolution reveals that cation disorder is nucleated at low co-ordination, high energy sites of the nanoparticle where cationic vacancy layers intersect with surface facets. Time-dependent evolution of the reciprocal lattice of individual nanoparticles shows that the initiation of cation disorder is accompanied by a ~3% compression of the anionic lattice, establishing a correlation between these two structural features of the lattice. The spatiotemporal insights gained here advance understanding of order-disorder transitions, ionic structure and transport, and the role of nanoparticle surfaces in phase transitions.

## Abstract (web summary)

Solid-solid phase transitions are processes ripe for the discovery of correlated atomic motion in crystals. Here, the authors monitor an order-disorder transition in real-time in a single nanoparticle of the super-ionic solid, cuprous selenide, using in-situ high-resolution transmission electron microscopy.

## Introduction

The advent of in-situ high-resolution transmission electron microscopy (HRTEM) has made available a tool for monitoring solid-state phase transitions and elucidating their underlying atomistic dynamics^1–4^. In-situ HRTEM has been exploited to gain a deeper understanding of the crystallization and growth of nanoparticles^5,6^ and post-synthetic transformations of nanoparticles, such as hydrogenation^7^. Here, we use in-situ HRTEM to follow an order–disorder phase transition with atomic resolution.

The transition we investigate is central to fast-ion conducting solids, which have potential as solid electrolytes for Li-ion batteries^8–15^. Solids in this class are crystalline at room temperature; but at elevated temperatures, they exhibit a disordered cationic sub-lattice with high cation mobility. As our model system, we choose cuprous selenide (Cu_2_Se or Cu_2−x_Se), a common fast-ion conductor^16–22^. The choice was motivated by the structural features of Cu_2_Se. A fraction of the Cu^+^ in Cu_2_Se is displaced from the ideal tetrahedral sites within the near-face-centered cubic Se^2−^ sub-lattice cage^21,23–25^. The resulting tetrahedral-site Cu-vacancies order to form a super-lattice^16,19,21,26–28^ with a characteristic inter-vacancy-layer spacing of ~0.68 nm. But, at temperatures greater than ~140 °C in bulk Cu_2_Se, the Cu^+^ becomes mobile over multiple types of interstitial sites and the vacancy ordered (VO) structure^23,25,26^ is lost. The resulting superionic (SI) phase of Cu_2_Se exhibits fast-ion conduction^19,25^. We are motivated to study the temperature-induced transition from the ordered phase to the SI phase, because newer understanding can lead to strategies for enhancing ion transport and reducing the temperature needed for achieving fast-ion conduction and bringing it closer to the operating temperature range of Li-ion batteries.

Using in-situ HRTEM, we watch the VO-to-SI transition within a single Cu_2−x_Se nanoparticle, providing us a unprecedented look at the spatiotemporal progression of the phase transition. We identify the precise atomic site where cation disordering is initiated and uncover correlated structural motions involved in the process.

## Results

### Nanoparticles with an order–disorder transition

**Fig. 1:**
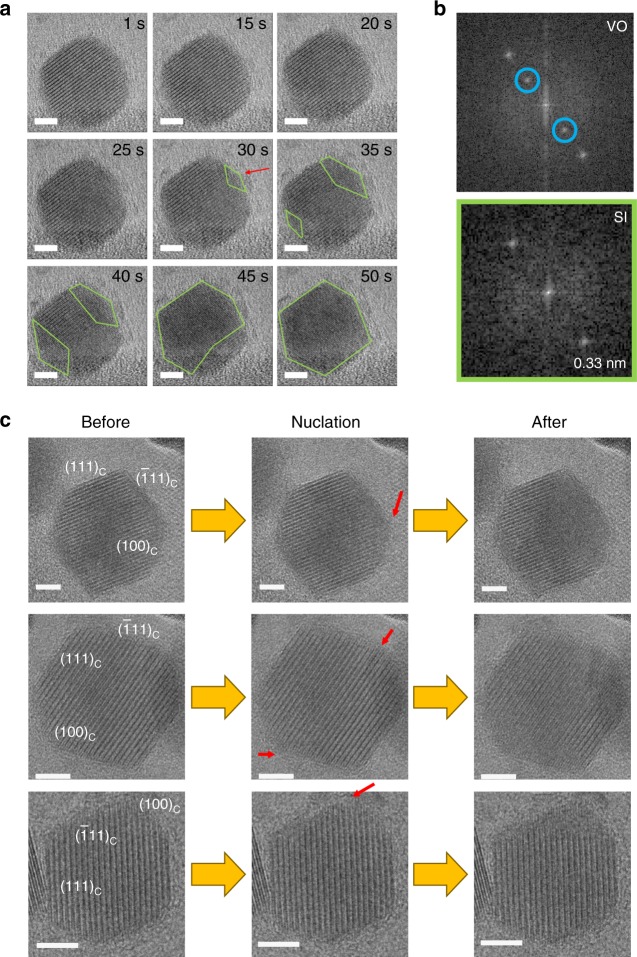
HRTEM imaging of the order–disorder phase transition in Cu_2−x_Se HNPs. **a** Selected movie frames from continuous HRTEM imaging of an HNP undergoing a transition from the vacancy-ordered (VO) phase to the superionic (SI) phase. The time relative to the start of the movie is indicated for each frame. Before t = 30 s, there is nucleation of a cation-disordered region at the location labeled by the red arrow. The SI phase (marked by green outlines) grows across the HNP over time. **b** FFT of the VO region (top) and SI region (bottom) of the HNP, obtained from the t = 35 s frame shown in Fig. 1a. The VO phase shows characteristic vacancy super-lattice spots (*d* ~ 0.68 nm, marked by blue circles) in the FFT, which are absent for the SI phase. **c** HRTEM snapshots of an HNP before, around, and after the point of nucleation of the order–disorder transition. Corresponding FFTs are shown in Supplementary Fig. 3. This evolution is shown for three different HNPs (one per row). The nucleation site, where vacancy-ordering is first observed to be lost, is marked by a red arrow in each case. This site was identified on the basis of a difference in the local lattice fringe pattern in the before and after frames. It is found that nucleation occurs at a vertex site at the intersection of ($\bar 1$11)_c_ and (100)_c_ surface facets. These surface facets (labeled in the leftmost image of each row) were assigned on the basis of FFT analysis of the HRTEM images of individual HNPs, as described in Supplementary Fig. 4. Scale bars in images are 5 nm in length

We filmed using in-situ HRTEM individual Cu_2−x_Se nanoparticles as they underwent a transition from the VO phase to the SI phase (Fig. 1, Supplementary Movies 1 and 2). The nanoparticles have a hexagonally shaped cross-section with a mean size of ~22 nm measured along the diagonals of the hexagon (Supplementary Fig. 1). At this relatively large size, the hexagonal nanoparticles (HNPs) have a phase transition temperature of ~140 °C similar to that of the bulk solid^23^. But, unlike the bulk solid, surface facets comprise an important feature of the HNPs. We refer to the HNPs using the sub-stoichiometric Cu_2−x_Se formula simply to account for a small amount of air oxidation that the HNPs undergo in the course of their transfer to the HRTEM instrument. However, the small degree of sub-stoichiometry, *x*, does not cause any deviation in the lattice structure of these HNPs relative to that of stoichiometric Cu_2_Se. Prior to any transition, (Fig. 1), the HRTEM images of the HNPs show the characteristic lattice fringe pattern of low-temperature Cu_2_Se. A lattice fringe pattern with a period of ~0.68 nm is observed along the [111]_c_ crystallographic axis of the pseudo-cubic (c) unit cell. This pattern is a manifestation of a Cu-vacancy super-lattice formed by the arrangement of Cu-vacancies once every fourth Cu^+^ layer along [111]_c_^29^.

### In-situ imaging of order–disorder phase transition

The temperature-dependent phase transition in an HNP was induced by the electron beam, which heats up the HNP^3,30^. From the estimates of the local temperature achieved under the electron beam irradiation conditions of our studies (see Supplementary Note), it is plausible for beam-induced heating to raise the temperature of an HNP to a value above the phase transition temperature of ~140 °C. As the beam-induced phase transition progressed, a high-resolution movie of the HNP at 1 frame per s was acquired. Select snapshots from such a movie of a representative HNP are shown in Fig. 1a. As shown by the example, the HNPs start in the VO phase and eventually transition to the SI phase under the focused electron beam. The formation of the SI phase is seen as a loss of the ordered Cu-vacancy super-lattice of ~0.68 nm periodicity, as also confirmed by the fast-Fourier transforms (FFTs) of VO and SI regions in the real-space image (Fig. 1b). In the SI phase, lattice fringes are observed with a periodicity of ~0.33 nm, which is the characteristic Se^2−^–Se^2−^ interlayer spacing along [111]_c_^16,26^. In other words, the Cu^+^ sub-lattice, being disordered, does not contribute to the lattice pattern.

### Precise site for nucleation of cation disorder

As seen from Fig. 1a, at intermediate stages in the transition, domains of the SI and VO phases co-exist in the HNP. With increasing time, the SI domain grows in size and the VO domain shrinks until the HNP is fully in the SI phase. We used the characteristic lattice fringe spacing d_[111]c_ to identify the portions of the HNP that were in the VO (d_[111]c_ ≈ 0.68 nm) and SI (d_[111]c_ ≈ 0.33 nm) phases. In this manner, we were able to obtain spatial maps (Fig. 1c) and time-trajectories (Supplementary Fig.  2) of the growth of the SI phase in a single HNP as a function of time.

**Fig. 2:**
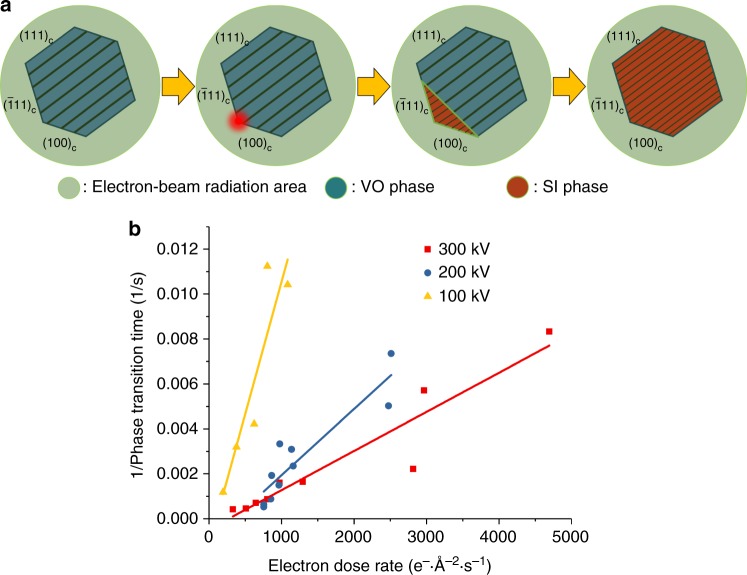
Spatiotemporal progression of the order–disorder transition. **a** Schematic of the electron-beam induced transition of the Cu_2−x_Se HNPs from the VO phase to the SI phase. **b** The speed of the phase transition as a function of the electron dose rate at three different accelerating voltages. The phase transition speed is defined by the inverse of the time it takes from the start of the acquisition for the entire Cu_2−x_Se HNP to transition into the SI phase. Each data-point is obtained from a separate single HNP subject to electron-beam irradiation of a fixed electron dose rate at a fixed accelerating voltage. Solid lines are meant to guide the eye

We found that the phase transition was invariably initiated at the surface of the HNP, specifically at a vertex site (Fig. 1c and Supplementary Fig. 3), a feature observed for 14 of 15 Cu_2−x_Se HNPs were studied. A similar phenomenon has been observed for hydrogen intercalation in faceted α-palladium hydride nanoparticles^31^. Vertex sites on a nanoparticle surface have a lower atomic co-ordination than other lattice sites, resulting in a higher local surface energy. As a result, the energy barrier for nucleation of the SI phase can be considerably lower at such sites. There is an alternate possibility: due to the smaller mass thickness at the vertex sites, the electron beam effect is pronounced at these locations, resulting in preferential nucleation at these sites. If this were the case, then nucleation would have been observed at any one of the six vertices of the HNPs. However, as seen for three representative HNPs in Fig. 1c, the transition nucleated at only the specific vertex, or pair of vertices, which lie at the intersection of the ($\bar 1$11)_c_ and (100)_c_ surface facets (Supplementary Fig. 4). There is a possible reason why this specific pair of vertices is the favored location for nucleation of cation disorder. These vertices are located normal to the direction along which Cu-vacancy planes are oriented. The vacancy planes intersect these vertices, reducing the local coordination number and increasing surface energy even further. In essence, we find that the phase transition is initiated at the site of confluence of surface atoms and Cu-vacancies, where atomic co-ordination is likely to be the weakest. The spatial manner in which the SI phase nucleates and grows across the Cu_2−x_Se HNP is depicted by the schematic in Fig. 2a.

### Electron beam effects involved in the phase transition

Since the electron beam is employed not only for imaging but also for inducing the transition, we characterized electron beam effects in more detail and examined the role of multiple effects. In general, electron beam irradiation can influence a specimen in two ways: via elastic scattering effects such as knock-on damage^30^ and inelastic scattering effects such as specimen heating and electrostatic charging^32,33^.

We studied the influence of electron dose rate and accelerating voltage on the phase transition speed (Fig. 2b). The transition speed was defined as the inverse of the time required for the completion of a phase transition in an HNP. The higher the electron dose rate, at a constant accelerating voltage, the higher was the speed of the transition. At a similar electron dose rate, the transition speed was lower for higher accelerating voltages. These trends indicate that inelastic scattering processes are responsible for the electron beam-induced phase transition. We can further rule out that the phase transition is induced by the knock-out displacement of Cu because this process requires a critical accelerating voltage of 420 kV^30^, which is higher than the accelerating voltages used in our studies. Even though electrostatic charging of the HNP cannot be ruled out, these results, combined with the estimated local temperature of our electron beam irradiation, indicate that beam-induced heating is the primary means for inducing the VO-to-SI phase transition. Consistent with this interpretation, the higher the electron dose rate, the higher is the temperature reached within the beam-irradiated HNP (Supplementary Note), and faster is the transition. In effect, the electron beam parameters can be employed as handled for controlling the phase transition kinetics.

The beam-induced phase transition was also found to be reversible (Supplementary Fig. 5a). When an HNP that had fully transitioned into the SI phase was allowed to stand in the absence of electron beam irradiation, the HNP reverted to the VO phase. Thus, it appears that the HNP does not suffer any irreversible damage or structural changes due to electron beam irradiation. In the absence of beam irradiation, the HNP presumably returns to a temperature below the phase transition temperature, resulting in the recovery of the low-temperature VO phase. We also observed that if the electron beam irradiation was interrupted while an HNP was in the midst of a phase transition, the HNP began to revert back to the VO phase (Supplementary Fig. 5b). Thus, beam-induced heating appears to be needed not only for initiating the transition but also for maintaining the higher temperature needed for the SI phase to extend across the entire HNP. Thus, it is possible to control the phase of the HNP by modulation of the electron-beam irradiation, with possible applications in electron-beam writing of information at high-densities. Information encoded in the form of the phase can be read out at low electron beam dose rates: for instance, at a dose rate below 147 e^−^ Å^−2^ s^−1^ at 300 kV, no phase transition was observed for at least 30 min and the HNP remained in the VO phase.

### Kinetics and dynamics of the phase transition

**Fig. 3:**
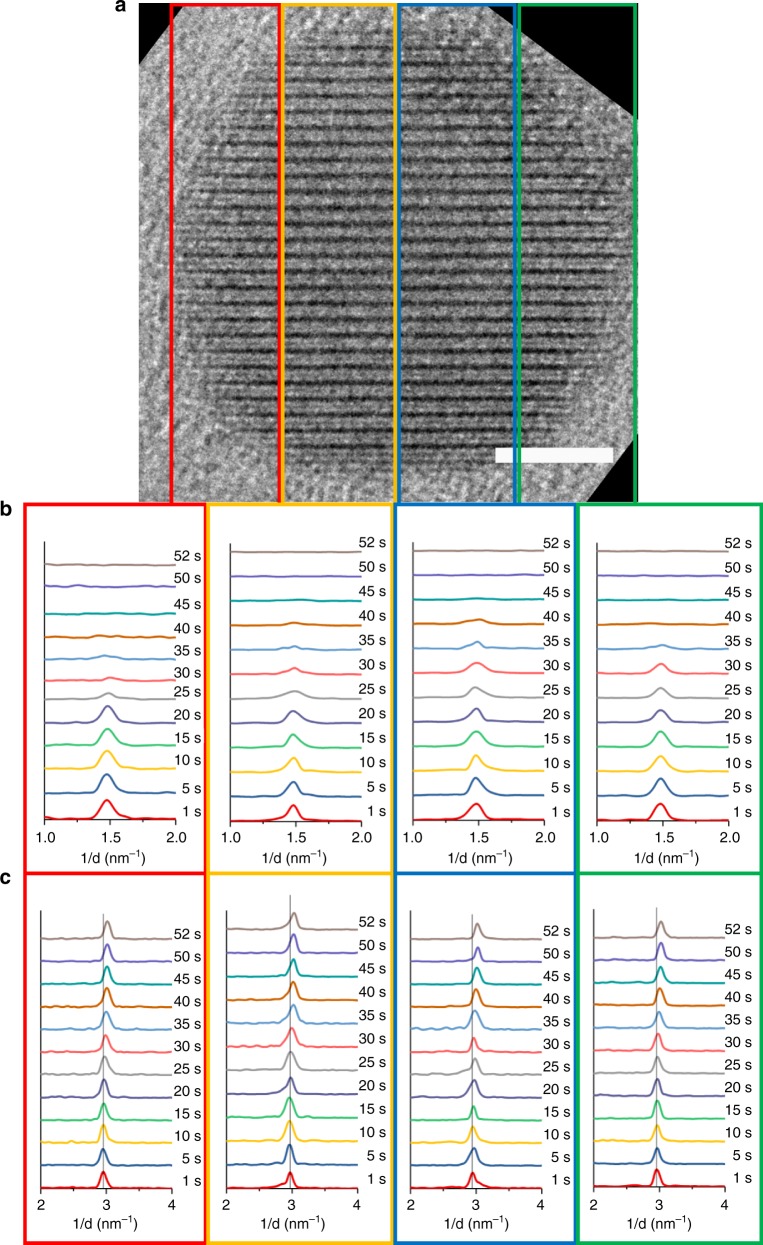
Se^2−^ sub-lattice undergoes compression alongside the loss of vacancy ordering. **a** HRTEM image, with a 5-nm scale bar, of an HNP oriented with the [111]_c_ direction aligned vertically. This is the same HNP as the one shown in Fig. 1a. The oriented HNP lattice is sub-divided into four distinct regions, which are outlined by the four colored boxes. **b** The time-evolution of the PDFs in the *d*^−1^ = 1–2 nm^−1^ range for each of the four regions in the corresponding column. Every PDF was normalized by dividing by the maximum of the PDF with the highest intensity peak in that series. **c** The time-evolution of the PDFs in the *d*^−1^ = 2–4 nm^−1^ range for each of the four regions in the corresponding column. Every PDF was separately normalized from 0 to 1. The PDFs are stacked vertically as a function of the time-point in the movie. The vertical line is aligned with the maximum of the reciprocal-lattice peak at the starting time-point. For the 1 s and 52 s frames, the reciprocal-lattice peak around 3 nm^−1^, which corresponds to the Se^2−^–Se^2−^ interplanar spacing along [111]_c_, was fitted with a Gaussian function to yield the *d*-spacing, which was found to be 0.34 nm (SD = 0.34%) and 0.33 nm (SD = 0.10 %), respectively. The reported *d*-spacing is an average over the four regions and the % standard deviation is indicated in the parentheses. From the PDFs, it is seen that the Se^2−^–Se^2−^peak shifts to smaller *d*-spacings alongside the loss of the Cu-vacancy super-lattice peak around 1.5 nm^−1^. The same behavior is shown by all 10 HNPs arbitrarily selected for this analysis from the total available sample set of 15 HNPs (see Supplementary Fig. 6)

In addition to the identification of the atomic site of nucleation, the structural dynamics of the phase transition was also revealed by in-situ HRTEM. As described in a previous section, the SI phase of Cu_2−x_Se lacks the ordered super-lattice arrangement of the Cu^+^ sub-lattice that the VO phase exhibits. There is one additional structural difference: the overall unit cell is known to have a smaller volume^26^ in the SI phase as compared to the VO phase. We observed a signature of this lattice volume difference in the Se^2−^–Se^2−^ interplanar spacing along [111]_c_ measured from FFTs (Fig. 3c) of HRTEM snapshots. The starting HNPs exhibit a Se^2−^–Se^2−^ interplanar spacing of ~0.34 nm in the VO phase; whereas post-transition, the HNPs show a moderately compressed lattice with a Se^2−^–Se^2−^ interlayer spacing of ~0.33 nm, which is ~3% smaller than that in the VO phase.

From HRTEM movies of single HNPs, we were able to monitor the real-time evolution of the aforementioned structural features as the HNP transitioned from the VO phase to the SI phase. For this purpose, the HRTEM movies were subject to a frame-by-frame FFT analysis, which yielded pair distribution functions (PDFs) as a function of time (Fig. 3). Four different sections of an HNP (Fig. 3a) were separately analyzed to gain a spatially resolved perspective.

As the Cu^+^ sub-lattice becomes locally disordered in a section of the HNP, the reciprocal-lattice peak near 1.5 nm^−1^, which corresponds to Cu-vacancy ordering, decreases in intensity until the peak finally disappears when the transition to the disordered phase is complete in that section (Fig. 3b). Alongside, the time evolution of the Se^2−^ sub-lattice is manifested in the reciprocal-lattice peak near 3 nm^−1^, which corresponds to the Se^2−^–Se^2−^ interplanar spacing along [111]_c_. With increasing time, the peak shifts to higher reciprocal lattice spacing (*d*^−1^) or smaller *d*-spacing, indicating a compression of the lattice. What is most notable is that this lattice compression begins to occur prior to the lattice fully adopting the SI phase. This trend is observed for all four regions of the HNP shown in Fig. 3 and in 9 additional HNPs presented in Supplementary Fig. 6. This observation makes evident a correlated re-organization of the anionic and the cationic sub-lattices of Cu_2−x_Se in the order–disorder transition.

**Fig. 4:**
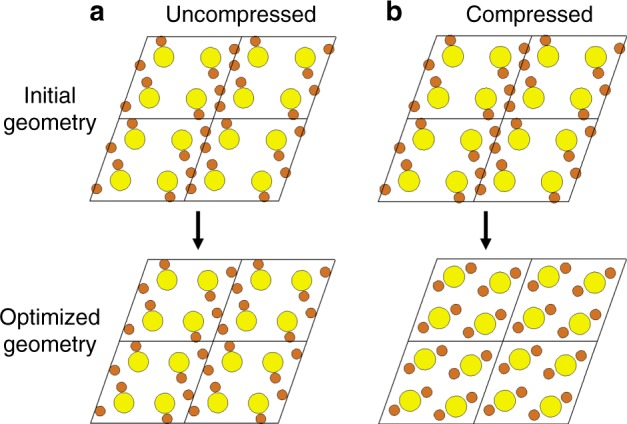
DFT investigation of the correlation between lattice strain and cation disorder. **a** In the absence of any strain, a Cu_8_Se_4_ unit cell maintains its Cu-vacancy order upon geometry optimization. **b** In contrast, a Cu_8_Se_4_ unit cell, subject to a 3.9% compressive strain, loses Cu-vacancy order upon geometry optimization. Se^2−^ is shown in yellow and Cu^+^ in orange. Note that 2 × 2 × 1 supercells are shown for easier visualization of the vacancy ordering. The black arrow signifies the geometry optimization procedure. The unit cell parameters for the two cases are described in the methods

Such a correlated structural re-organization is reproduced in density functional theory (DFT) computations (Fig. 4). Energy relaxation of a Cu_2−x_Se unit cell bearing a VO structure resulted in only minor displacement of the Se^2−^, whereas the Cu-vacancy ordering was fully preserved. However, a Cu_2−x_Se unit cell with a lattice compressed by 3.9% isotropically (including along [111]_c_) lost its ordered Cu-vacancy arrangement upon energy relaxation. This observation is consistent with a previous postulate that compressive strain stabilizes Cu^+^ occupancy of otherwise higher-energy octahedral sites^23,34^. When Cu^+^ gain access to these new interstitial (octahedral^24,25^ or other nearby^35^) sites, the Cu^+^ sub-lattice becomes mobile and the Cu-vacancy ordering is lost.

In conclusion, in-situ HRTEM imaging allowed atomic-level monitoring of an order–disorder transition in nanoparticles of Cu_2−x_Se, a model super-ionic conductor. We were able to resolve super-lattice ordered and cation-disordered domains within individual HNPs and follow the time-evolution of these domains over the course of the transition. Enabled by the atomic resolution, we identified the site where cation disorder is nucleated. The nucleation site is found to be located on the HNP surface, specifically at vertices where the atomic co-ordination is significantly lower than in the bulk due to the confluence of point defects (Cu-vacancies) with surface atoms. Secondly, the HRTEM movies reveal that the loss of Cu-vacancy ordering is accompanied by a lattice compression of the Se^2−^ cage. Such correlated motions exemplify the mechanistic richness of phase transitions. More specifically, we gain a deeper understanding of the cation disorder in Cu_2−x_Se, the control of which would enable high-performance solid-electrolytes^18,36^.

## Methods

### Cu_2−x_Se HNP synthesis

Cu_2−x_Se HNPs were synthesized on the basis of a published procedure^37^ with some modification. In a 50 mL, three-necked round-bottom flask, 2 mmol (0.198 g) of anhydrous CuCl was dissolved in a solvent mixture of 10 mL of oleylamine and 10 mL of 1-octadecene (ODE). The CuCl solution was heated to a temperature of 80 °C and subject to vacuum degassing for 2 h. The temperature was then raised to 330 °C under Ar flow. A fresh Se-ODE solution was prepared in the glove box by dissolving 1 mmol (0.078 g) of Se in 5 mL of ODE and heating the mixture to 200 °C for 20 min under stirring, followed by sonication for 10 min until the Se dissolved. At 330 °C under Ar flow, the Se-ODE solution was rapidly injected into the CuCl solution in the reaction flask. Upon addition of the Se-ODE solution, the temperature dropped. Following the recovery of the temperature to 310 °C, the reaction was allowed to proceed for 15 min. After this period, the reaction mixture was allowed to cool and then 10 mL of toluene was added to it. The reaction mixture was transferred to the glove box for oxygen and moisture-free processing. The reaction mixture was diluted with 5 mL of ethanol and subject to centrifugation at 20 × *g* for 5 min, for removal of unreacted precursors, which stay in the resulting supernatant. The precipitate containing Cu_2−x_Se nanoparticles and large Cu_2−x_Se nanoplatelets, capped with oleylamine ligands, was dispersed in 5 mL of toluene and subject to another round of purification by the addition of 1 mL of ethanol and low-speed centrifugation at 20 × *g* for 5 min. The supernatant from this procedure, which is relatively purified of large nanoplatelets, was subject to multiple cycles of centrifugation. The final precipitate of this procedure containing the Cu_2−x_Se HNPs was stored in the glovebox for further use. The HNPs were dispersed in toluene for further characterization.

### In-situ HRTEM imaging and analysis

For HRTEM imaging, samples were prepared by casting a few drops of the HNP colloid on a TEM substrate consisting of an ultrathin carbon on a lacey carbon film supported by a 400 mesh Cu grid (Ted Pella Co.). HRTEM imaging was performed on a Hitachi 9500 instrument equipped with an Orius charge-coupled device (CCD) camera. Unless otherwise noted, HRTEM was performed with an accelerating voltage of 300 kV and an electron beam current of 2 or 6 μA. Low-magnification images were acquired for surveying nanoparticle morphology, which show that the nanoparticles have a hexagonal shape (Supplementary Fig. 1).

For in-situ monitoring of the order–disorder transition within individual HNPs, continuous higher magnification HRTEM imaging of a fixed region containing well-isolated HNP or HNPs was performed. Movies were acquired at a rate of 1 frame s^−1^. The electron beam irradiation was kept uninterrupted during the course of the phase transition. HRTEM movie frames and their FFTs were analyzed using the software ImageJ to determine the time-evolution of the VO and SI phase regions within the HNP.

A separate set of studies was conducted to examine how the electron dose rate and accelerating voltage influence the ability to induce the phase transition and the effect of these parameters on the time required for completion of a phase transition. In each study, an individual HNP was subject to electron beam irradiation of a fixed electron beam dose rate at a fixed accelerating voltage and a movie was recorded. Several such individual HNP experiments were conducted at three different accelerating voltages (100, 200, and 300 kV) and several different electron dose rates. The latter was changed by variation of the condenser aperture and/or the brightness variable. Electron dose rate (EDR) was determined from the recorded exposure density at the phosphorescent screen as follows:

1 $${\mathrm{EDR}} \, ( {\mathrm{e}}^{-} {\mathrm{\AA}}^{ - 2} {\mathrm{s}}^{ - 1} ) = \frac{{\mathrm{Exposure}}\,{\mathrm{density}} ( {\mathrm{C}}\,{\mathrm{cm}}^{ - 2} ) \times {\mathrm{Magnification}}^{2} \times 10^{ - 16}\, {\mathrm{\AA}}^{ - 2}\,{\mathrm{cm}}^{2}}{{\mathrm{Exposure}}\,{\mathrm{time}} ( {\mathrm{s}}) \times 1.602 \times 10^{-19}\,{\mathrm{C}}\,{\mathrm{per}}\,{\mathrm{e}}^{-}}$$

Note that if an HNP did not show any sign of nucleation in a 30-min period from the start of the acquisition, then the acquisition was stopped and the HNP was recorded to undergo no transition at those conditions. For instance, at 300 kV, at an electron beam dose rate below 147 e^−^ Å^−2^ s^−1^, no nucleation of the SI phase was observed in the 30-min period. On the other hand, if nucleation was observed before the 30-min time-point, the electron beam irradiation was continued without interruption until the phase transition was complete. Movies from these latter HNPs comprise the data plotted in Fig. 2b.

### Analysis of phase-transition dynamics

HRTEM movie frames were re-oriented such that the [111]_c_ direction of the HNP lattice was aligned along the line-profile axis. It must be noted that in the course of continuous imaging, there is thermal drift of the region being imaged. We corrected movie frames for this drift using the ImageJ Java plugin “NMS_fixTranslation_ver1.ijm”^2^. This plugin requires that the same landmark spot be manually identified in each movie frame to serve as the reference point for drift correction. The rest of the correction is automated by the algorithm. The manual reference point selection can introduce an error. This error is estimated to be a few pixels, which is considerably smaller than the hundreds-of-pixels dimensions of the regions selected for the section-by-section FFT analysis. Following drift correction, the movie was cropped around the HNP of interest and the HNP was divided into four sections as shown in Fig. 3. For each of these four regions, a line profile across the lattice fringe pattern along [111]_c_ was obtained using the open-source macro “StackProfileData_code.txt”^38^. One-dimensional FFTs of these line profiles were obtained using OriginPro program. The time-evolution of these PDFs formed the basis of the kinetic analyses described in Fig. 3 and Supplementary Fig. 6.

### DFT simulations

Plane-wave DFT simulations of Cu_8_Se_4_ unit cells were performed on the Quantum Espresso suite^39^ using norm-conserving Se.pbe-hgh.UPF and Cu.pbe-d-hgh.UPF pseudopotentials, the Perdew-Burke-Ernzerhof functional^40^, a kinetic energy cut-off of 200 Ry for wavefunctions, a 0.019 Ry smearing of electronic occupations, and automatically generated 6 × 8 × 6 Monkhorst-Pack k-point grids. The geometry of the uncompressed cell (Fig. 4) was adapted from a reported β-Cu_2_Se geometry^41^ and had unit cell parameters of *a* = 7.453 Å, *b* = 44.322 Å, *c* = 6.880 Å, *α* = 90.00°, *β* = 70.62°, and *γ* = 90.00 °. The Se^2−^–Se^2−^ interlayer spacing along <111>_c_ was 3.52 Å. To introduce compressive strain, the lattice constants were decreased by 3.9%. The compressed unit cell had parameters of *a* = 7.162 Å, *b* = 4.153 Å, *c* = 6.612 Å, *α* = 90.00°, *β* = 70.62°, and *γ* = 90.00°, but the atomic coordinates, expressed in crystal vector units, were the same as those in the uncompressed unit cell. The Se^2−^–Se^2−^ interlayer spacing along [111]_c_ was 3.38 Å in the compressed geometry. The uncompressed and compressed unit cells were each subject to geometry optimization. The optimization procedure was accomplished by constraining Se^2−^ and allowing Cu^+^ to relax and then allowing both Se^2−^ and Cu^+^ to relax without constraints. The Se^2−^–Se^2−^ interlayer spacing along [111]_c_ in the geometry-optimized cells (3.51 Å and 3.37 Å for uncompressed and compressed cases, respectively) was not significantly altered by geometry optimization.

## Supplementary information

Supplementary Information

Description of Additional Supplementary Files

Supplementary Movie 1

Supplementary Movie 2

## Data Availability

All raw images and source data are available from the authors upon reasonable request.
