# Supplementary material for: In-situ electron microscopy mapping of an order-disorder transition in a superionic conductor
Source: Nat Commun. 2019 Apr 3;10:1505. doi: 10.1038/s41467-019-09502-5 (PMC6447557; doi:10.1038/s41467-019-09502-5)
Supplement: Supplementary file 1 — Supplementary Information [file 41467_2019_9502_MOESM1_ESM.pdf]

SUPPLEMENTARY INFORMATION

In-situ electron microscopy mapping of an order-disorder transition in a superionic conductor

*Jaeyoung Heo,[1] Daniel Dumett Torres,[2] Progna Banerjee,[3] and Prashant K. Jain[2,3,4,5]**

[1]Department of Materials Science & Engineering, University of Illinois at Urbana−Champaign, Urbana, Illinois, 61801, United States

[2]Department of Chemistry, University of Illinois at Urbana−Champaign, Urbana, Illinois 61801, United States

[3]Department of Physics, University of Illinois at Urbana−Champaign, Urbana, Illinois 61801, United States

[4]Materials Research Laboratory, University of Illinois at Urbana−Champaign, Urbana, Illinois 61801, United States

[5]Beckman Institute of Advanced Science and Technology, University of Illinois at Urbana−Champaign, Urbana, Illinois 61801, United States

**Corresponding Author: E-mail: jain@illinois.edu.*

Supplementary Note

Estimation of local temperature under electron-beam irradiation

We estimated the local temperature resulting from electron-beam irradiation of the $Cu_{2-x}Se$ hexagonal nanoparticle (HNP) supported on an ultrathin carbon film on lacey carbon. We estimated the maximum temperature rise in the carbon substrate due to energetic dissipation of the electron beam traveling through the thickness of the carbon film.[1] The electron beam spot at the substrate has a radius r_0 estimated from the known beam current i of 6 µA and the resulting current density j as:

$$r_0 = \sqrt{\frac{i}{\pi j}} \qquad (1)$$

The beam current density was assumed to be uniform across the entire beam spot. Heat is generated by dissipation of the electron beam in the carbon film within the cylindrical region of the beam, with radius r_0 and depth t corresponding to the thickness of the carbon film. The generated heat is transferred to the carbon film outside of this region by conduction through the cylindrical surface of area $S = 2\pi rt$. The heat travels through the carbon film over a distance R until it reaches the Cu bar, where the temperature is assumed to be at ambient (T_o = 298 K). For a 400-mesh Cu grid each grid square has a side length of 42 µm, so R = 21 µm for a beam spot centered within the grid square. Radiation of heat was ignored since it is not expected to be a substantial effect at a temperature in the range of a few hundred K. At steady-state, the heat generation rate is balanced by the heat transfer rate resulting in the following equation for $R > r_0$:

$$\pi {r_0}^2 \frac{j}{e}\frac{\Delta Q}{\Delta x}\rho t = -\lambda 2\pi r t \frac{\mathrm{d}T}{\mathrm{d}r} \qquad (2)$$

where T is the temperature at distance r from the center of the beam spot, e is the electronic charge of 1.6 x 10^{-19} C, ρ is density with a value of 2.26 g cm^{-3} for carbon, λ is the thermal conductivity with a value of 1.5 x 10^{-2} J K^{-1} cm^{-1} s^{-1} for carbon,[1] and $\frac{\Delta Q}{\Delta x}$ is the energy dissipated per unit mass thickness. The steady-state temperature within the irradiated region is the highest at the center of the beam spot and obtained by solving Supplementary eq. 2:

$$T_{\mathrm{max}} = T_0 + \frac{j\rho}{2e\lambda}\frac{\Delta Q}{\Delta x}{r_0}^2 \ln\frac{R}{r_0} \qquad (3)$$

The energy dissipated per unit mass thickness in units of eV cm^2 $µg^{-1}$ was calculated as follows:

$$\frac{\Delta Q}{\Delta x} = 7.8 \times 10^4 . \frac{Z}{A} . \frac{1}{E} . (\ln\frac{E}{J}) \qquad (4)$$

where Z is the atomic number which is 6 for carbon, A is the atomic mass number which is 12 for carbon, E is the electron energy which is 3 x 10^5 eV, and $J \approx 13.5Z$ is the mean ionization energy. From Supplementary eq. 4, we obtained $\Delta Q/\Delta x$ = 1.0682 eV cm^2 $µg^{-1}$ within the carbon film.

Since the carbon film acts as a large heat bath for the relatively smaller $Cu_{2-x}Se$ HNP, the HNP will

be at a similar or higher temperature than the carbon film. Therefore, for an HNP located at the center of the beam spot, T_{max} provides a lower estimate for the temperature of the HNP under beam irradiation of a specific current density *j*. For the range of current densities (*j* = 1–7 A cm^{-2}) and a beam current of 6 μA used in our studies, we estimated T_{max}, which is tabulated below. The electron dose rate (EDR) corresponding to each current density is calculated as:

$$\text{EDR (e}^{-}\text{ Å}^{-2}\text{ s}^{-1}) = \frac{\mathbf{j\,(A\,cm^{-2})\,x\,10^{-16}\,Å^{-2}\,cm^{2}}}{\mathbf{1.602\,x\,10^{-19}\,C\,per\,e^{-}}} \quad (5)$$

and provided in the second column.

j (A cm^{-2})	EDR (e^{-} Å^{-2} s^{-1})	T_{max} (K)	T_{max} (°C)
1	624	362	89
2	1248	416	143
3	1873	447	174
4	2497	469	196
5	3121	486	213
6	3745	500	227
7	4370	512	239

The precise temperature at the HNP will depend on its location relative to the center of the beam spot, where the local temperature is the highest. The further away the HNP from the center, the lower will be its temperature. The local temperature decays to a value of T_0 = 298 K at a distance *R* from the beam spot.

Supplementary Figures

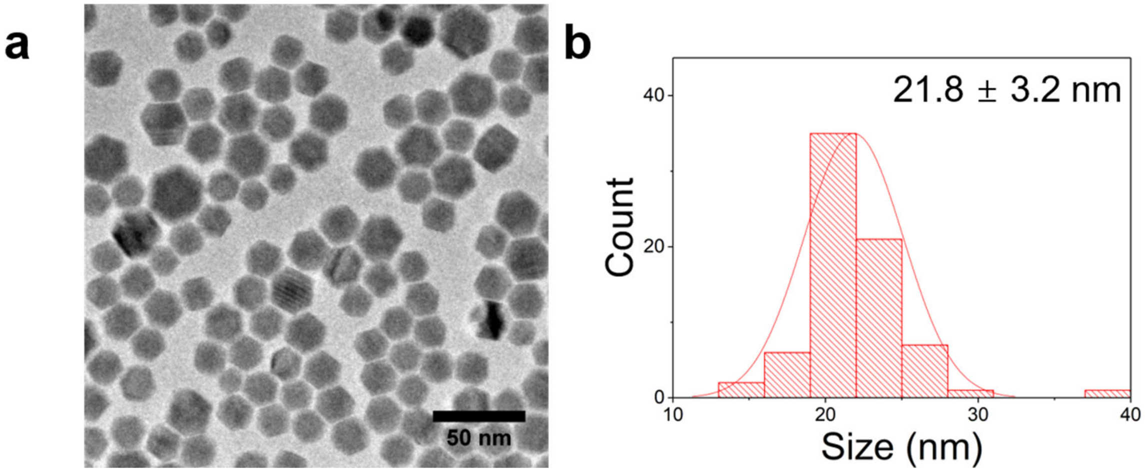

Supplementary Figure 1. Morphology characterization of $Cu_{2-x}Se$ HNPs by transmission electron microscopy (TEM). (a) A low-magnification (60.4 k×) TEM image of the $Cu_{2-x}Se$ HNPs. (b) A histogram showing a size distribution for the HNPs, along with a normal distribution fit. The size indicated is that measured along the diagonal of the hexagon, obtained by averaging measurements along two diagonals. The mean size is 21.8 nm with a standard deviation (SD) of 3.2 nm. We studied $Cu_{2-x}Se$ HNPs of this single-peaked, relatively narrow size distribution, so it was not possible to reliably study how size influences the phase transition kinetics; however, such a size-effect may be worthy of study in the future.

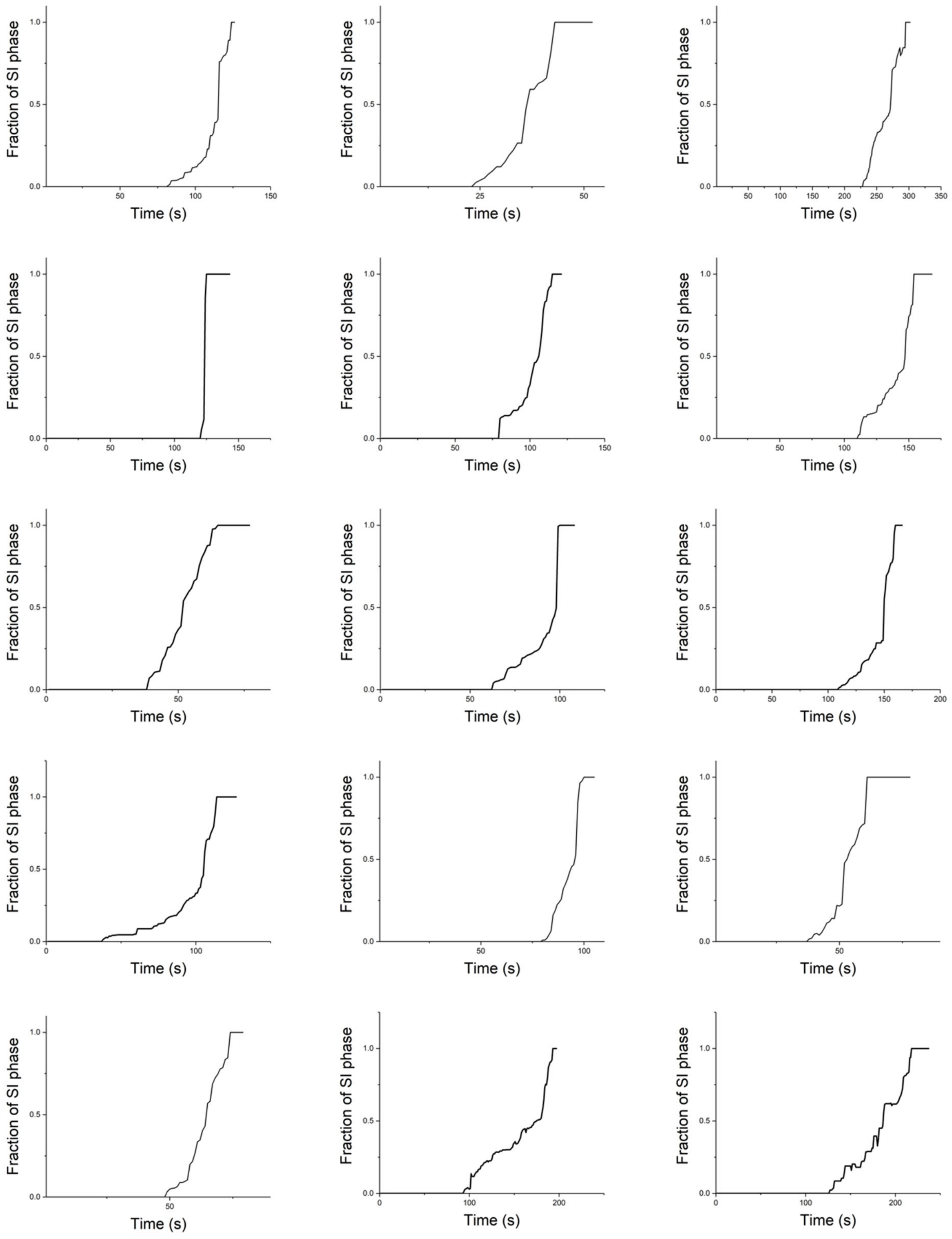

Supplementary Figure 2. For 15 different HNPs, the time-trajectory of the growth of the

superionic (SI) phase obtained from their continuous high-resolution transmission electron microscopy (HRTEM) video imaging, is shown. At each time-point, the area fraction of the SI phase was determined by identification of the vacancy ordered and disordered portions of the HNP in the HRTEM frame corresponding to the time-point. It must be noted that the electron beam dose rate was not controlled to be the same for all 15 HNP experiments. A separate study (Fig. 2b) was conducted with controlled electron beam dose rates and three different accelerating voltages (100, 200, and 300 kV) to determine how the phase transition speed is influenced by the electron beam dose rate and accelerating voltage.

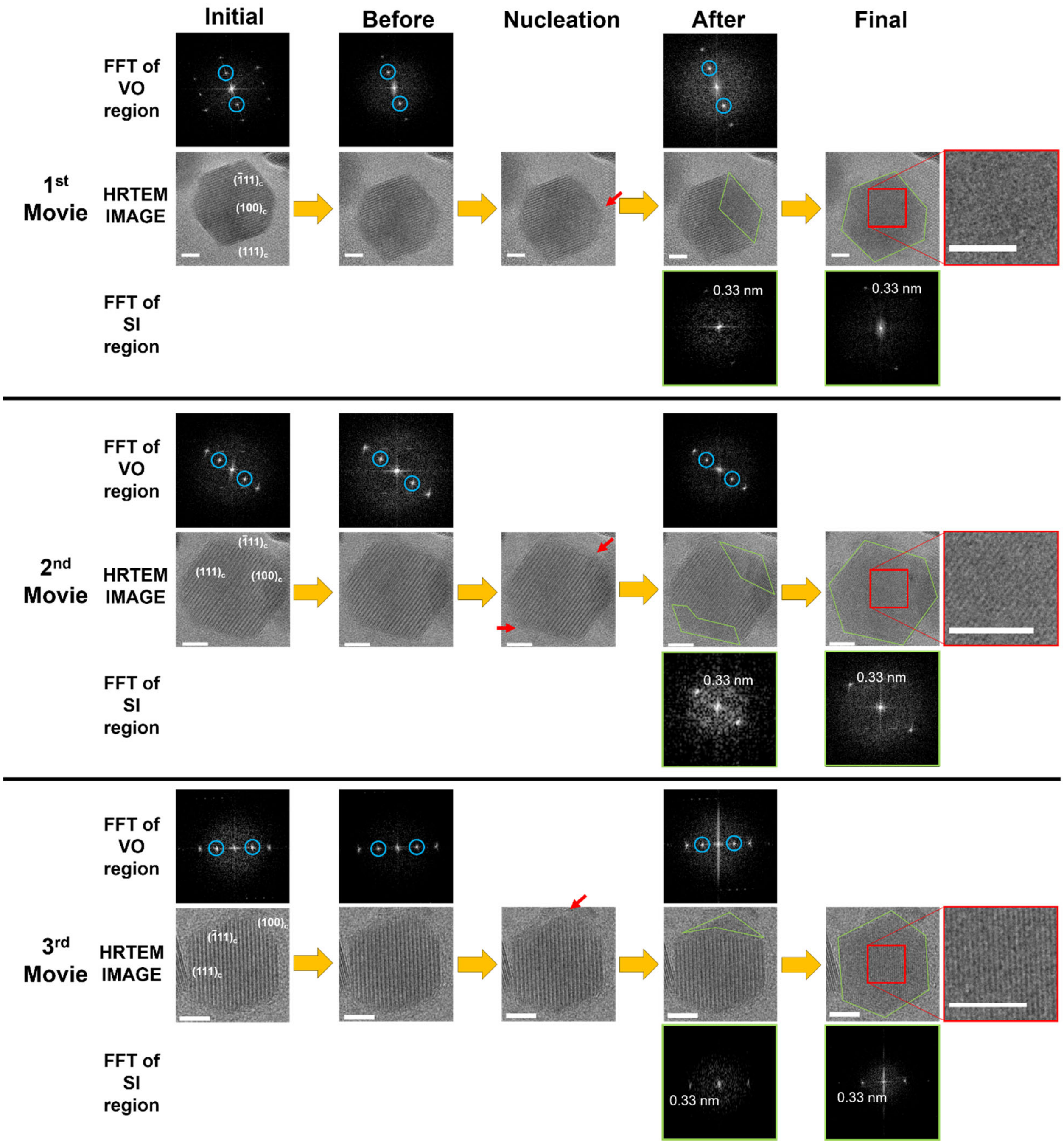

Supplementary Figure 3. Snapshots from movies showing the evolution of the real-space image and corresponding fast-Fourier transform (FFT) in the course of the phase transition for the three HNPs presented in Fig. 1c. Snapshots are shown left-to-right for multiple stages: *initial* frame of the movie, a time-point *before* the nucleation of the super-ionic (SI) phase, around the time-point of *nucleation*, a time-point *after* nucleation, and at the point where the phase transition is *final*. In the initial frame, the characteristic lattice fringes resulting from the vacancy-ordered (VO) phase

can be seen throughout the HNP. This lattice fringe pattern is completely lost in the final snapshot, signifying that the SI phase extends across the entire HNP. The latter is confirmed by the magnified image (rightmost column) of a select section (marked by the red box) of the HNP. In the interim stages, the SI phase (regions enclosed by green borders) is seen to nucleate and grow across the HNP. This is verified by FFTs selectively obtained from the VO region (shown above corresponding HRTEM image) and the SI region (shown below corresponding HRTEM image). The characteristic reciprocal lattice points for the vacancy super-lattice ($d \sim 0.68$ nm, marked by blue circles) are observed in the FFTs for the VO regions but lacking for the SI regions. The nucleation site, where vacancy-ordering is first observed to be lost, is marked in the HRTEM image by a red arrow for each HNP. The surface facets labeled in the *initial* HRTEM image were assigned on the basis of FFT analysis of the HRTEM images of individual HNPs, as described in Supplementary Figure 4. The scale bars in all images are 5 nm in length.

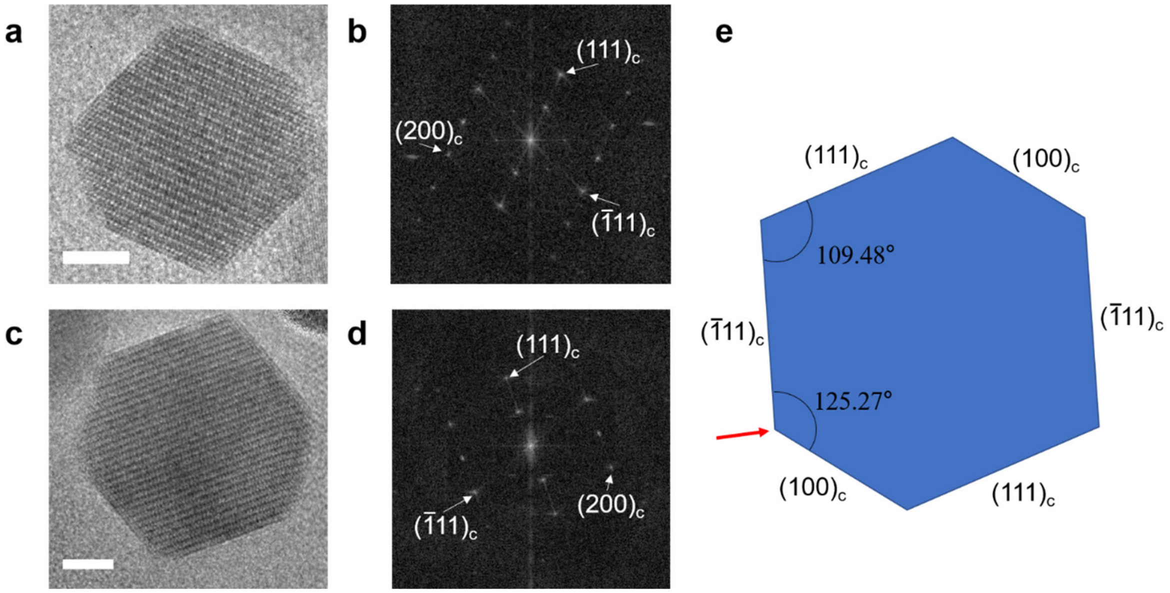

Supplementary Figure 4. Surface facets of $Cu_{2-x}Se$ HNPs. For two representative HNPs (a and c) an HRTEM image is shown. Scale bars are 5 nm in length. The lattice fringes are well-resolved all the way up to the surface facets of the HNP. Corresponding fast-Fourier transforms (FFT) of the HRTEM images in (a and c) are shown in (b and d), respectively. (e) A sketch of the in-plane projection of an HNP showing the surface facet construction and internal angles, which sum up to 720 °. Of the six facets, two are normal to the $[111]_c$ direction, along which the vacancy-related fringes are observed, are $(111)_c$ facets. Based on the cubic reduction of the unit cell and the fact that the included angles in the hexagon are close to 120 °, the likely assignments for the remaining four facets are one of $(100)_c$, $(\bar{1}11)_c$, $(\bar{1}12)_c$, $(\bar{1}22)_c$, or $(\bar{1}23)_c$. The labeled reciprocal-lattice spots in the FFTs in (b) and (d) correspond to inter-planar distances of 0.29 nm, 0.33 nm, and 0.34 nm, which correspond to $(200)_c$, $(\bar{1}11)_c$, planes, and $(111)_c$ planes, respectively. Therefore, the other two surface facets are $(100)_c$ and $(\bar{1}11)_c$ facets. This assignment is consistent with the facts that the angle between $(111)_c$ and $(100)_c$ planes is 125.27 °, the angle between $(111)_c$ and $(\bar{1}11)_c$ planes is 109.48 °, and that the sum of all included (in-plane) angles is 720.0 °. The vertices where the $(\bar{1}11)_c$ and $(100)_c$ facets intersect are sites for nucleation of cation disorder. One of these vertices is marked by a red arrow. Supplementary Figure 4c shows the same HNP as that in the top row of Fig. 1c and that in the middle row of Supplementary Figure 3.

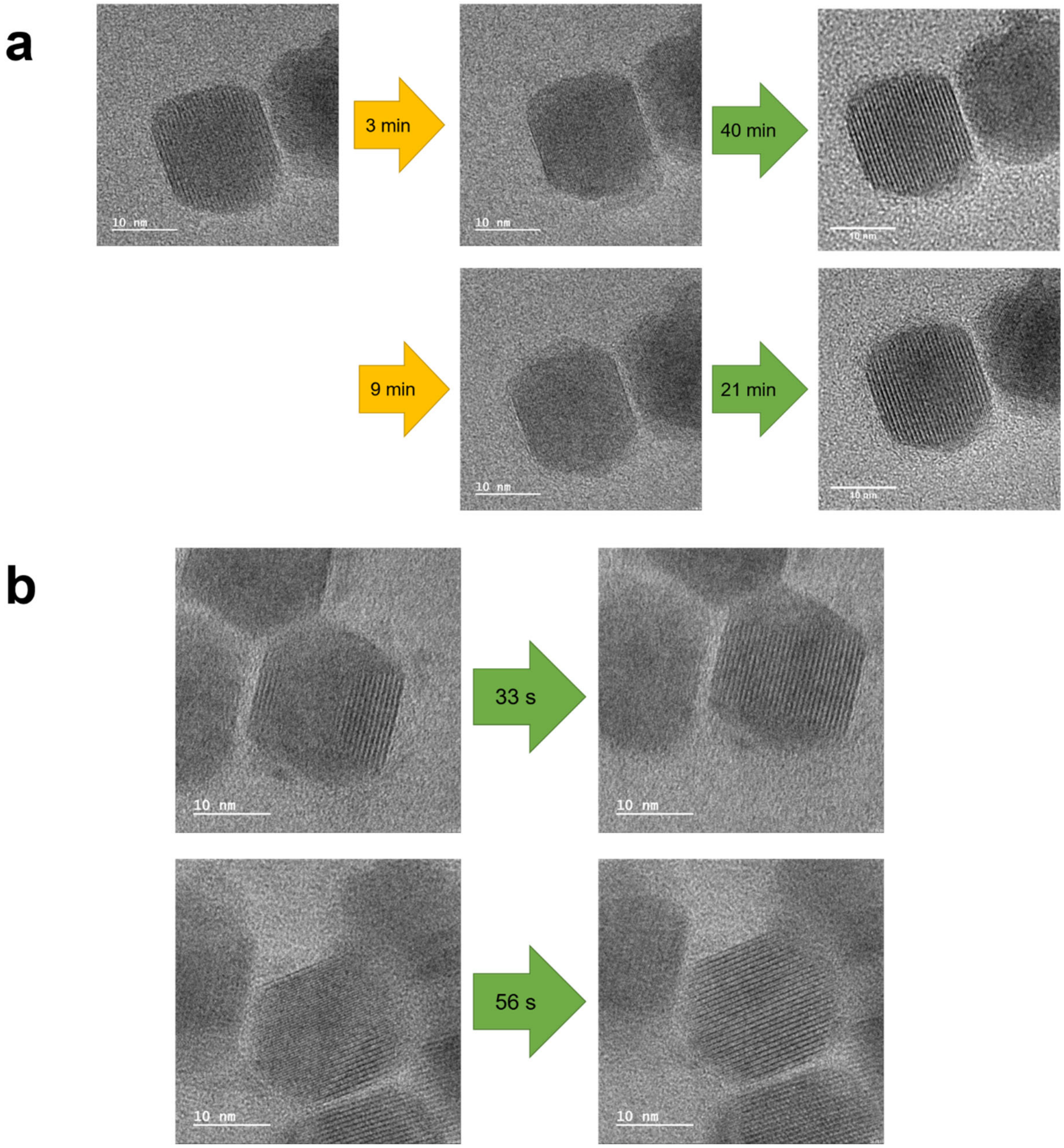

Supplementary Figure 5. (a) The VO-to-SI phase transition in a $Cu_{2-x}Se$ HNP was found to be reversible in a two-cycle test. The top row shows the first cycle and the bottom row shows the second cycle. Under continuous electron beam irradiation (yellow arrow), the HNP underwent the phase transition, which was completed in 3 min. The electron beam was then shuttered off and the HNP was allowed to stand in the "dark" (green arrow). Every few minutes, a single HRTEM snapshot (for minimizing beam exposure) was acquired, from which the HNP was found with time

to recover to the VO phase. In a snapshot taken after 40 min in the "dark", the HNP phase was found to be fully in the VO phase. Then the HNP was subject to a second cycle of continuous electron beam irradiation (yellow arrow). The HNP transitioned into the SI phase, a process which was completed in 9 min. Thereafter, the electron beam was shuttered off and the HNP was allowed to stand in the "dark" and found to fully recover to the VO phase in a snapshot taken after 21 min in the "dark". (b) For two different $Cu_{2-x}Se$ HNPs (top and bottom row), the electron beam irradiation was shuttered off while the VO-to-SI phase transition was ongoing in the HNP. The left column shows the HRTEM frame right before the beam was shuttered off. In this frame, the HNP is observed to be partly in the SI phase, while the rest remains in the VO phase. The electron beam was kept shuttered off for a time of tens of seconds (green arrow). When the beam irradiation was turned back on, the HNP was found to have largely reverted to the VO phase, as shown by the HRTEM snapshot in the right column.

a

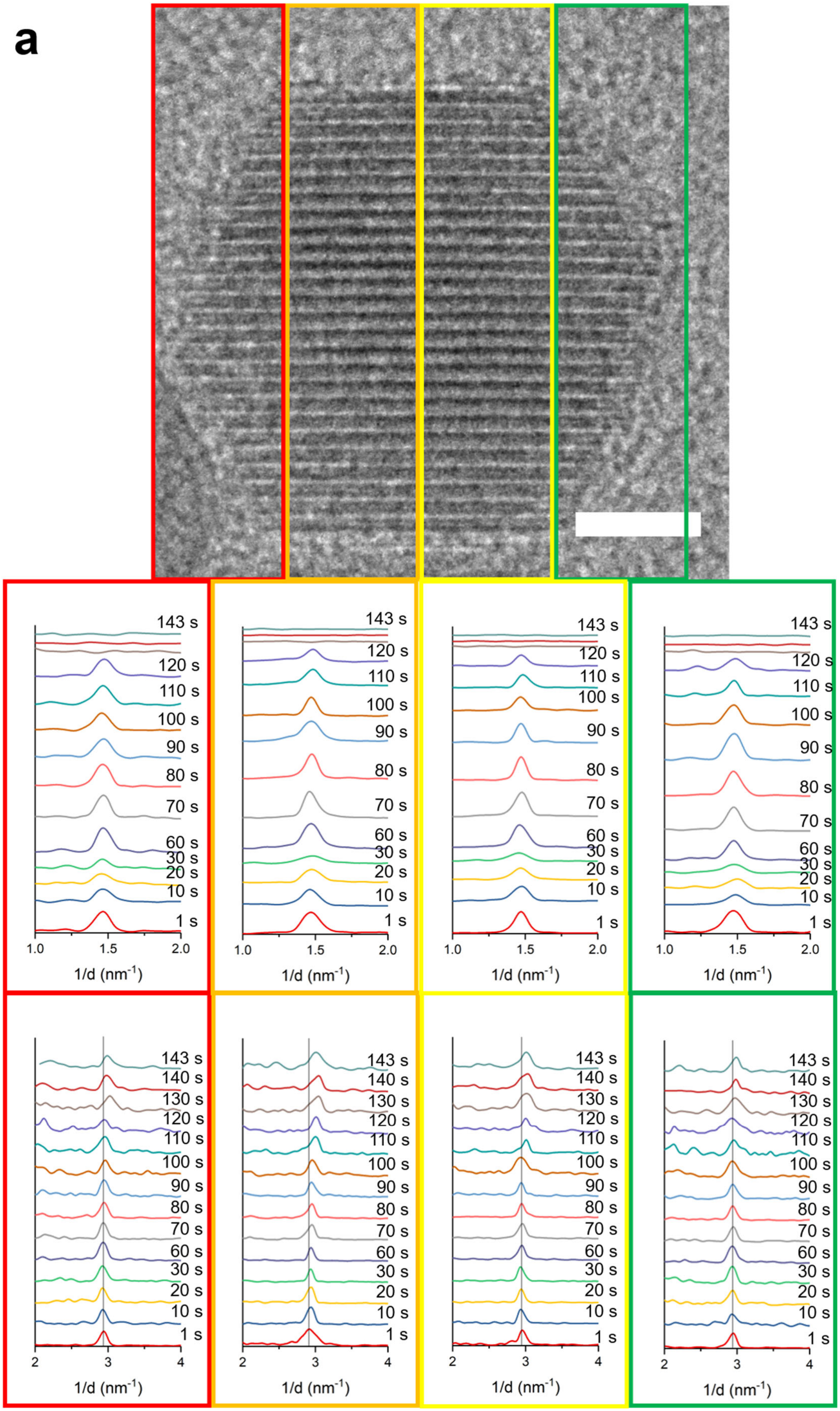
143 s
120 s
110 s
100 s
90 s
80 s
70 s
60 s
30 s
20 s
10 s
1 s
1/d (nm-1)
140 s
130 s

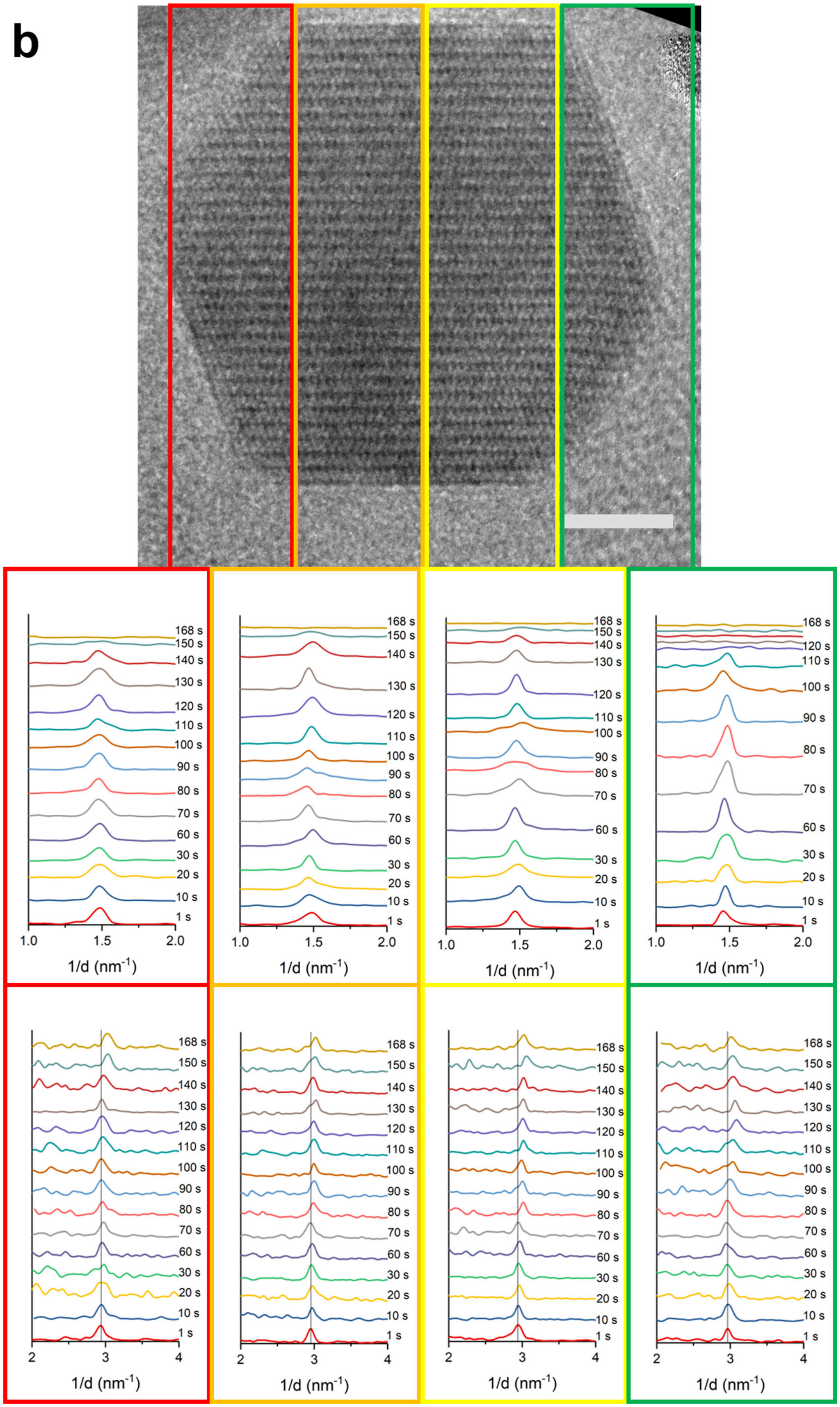

b
168 s
150 s
140 s
130 s
120 s
110 s
100 s
90 s
80 s
70 s
60 s
30 s
20 s
10 s
1 s
1.0
1.5
2.0
1/d (nm^{-1})
2
3
4

c

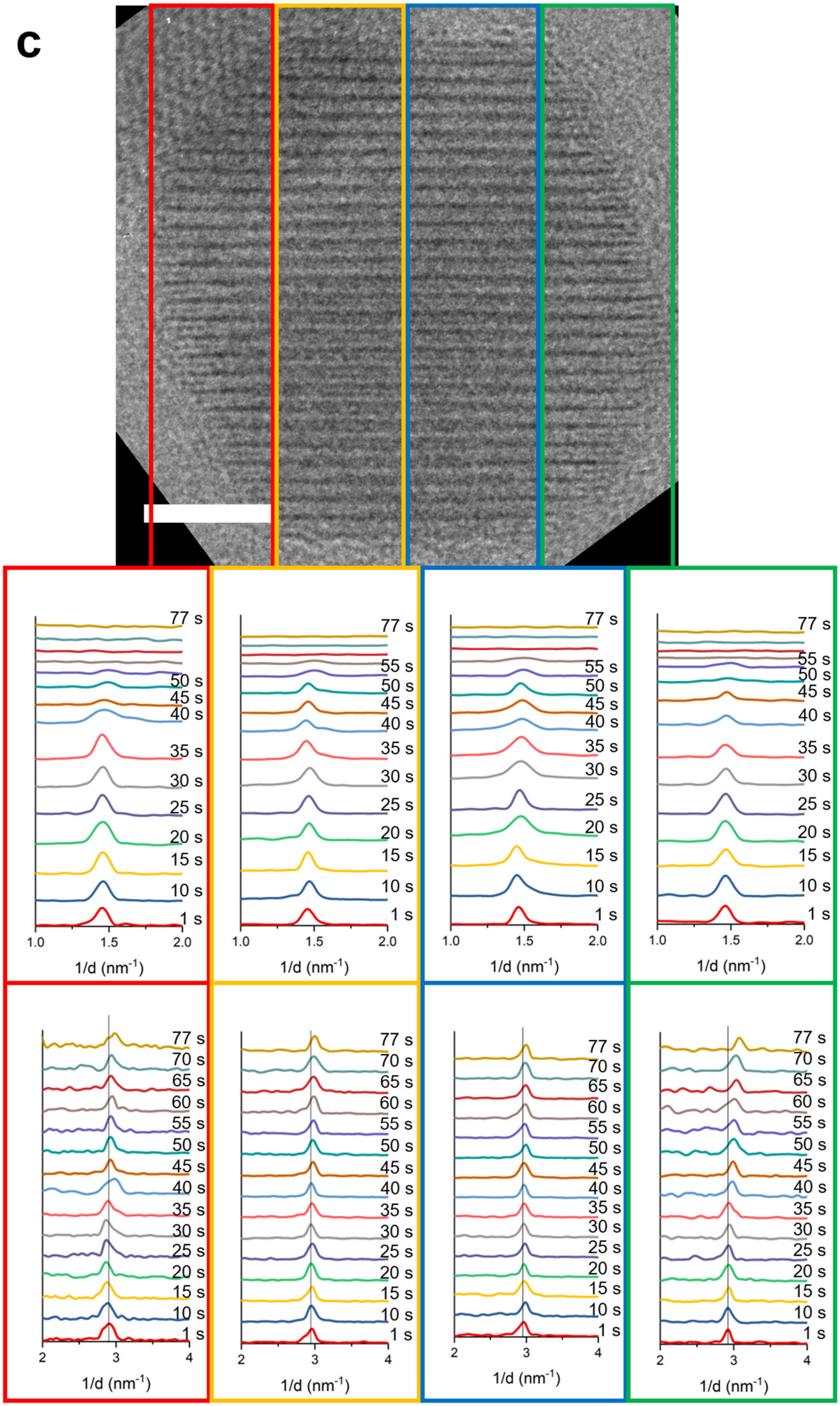
77 s
70 s
65 s
60 s
55 s
50 s
45 s
40 s
35 s
30 s
25 s
20 s
15 s
10 s
1 s
1.0
1.5
2.0
2
3
4
1/d (nm^{-1})

d

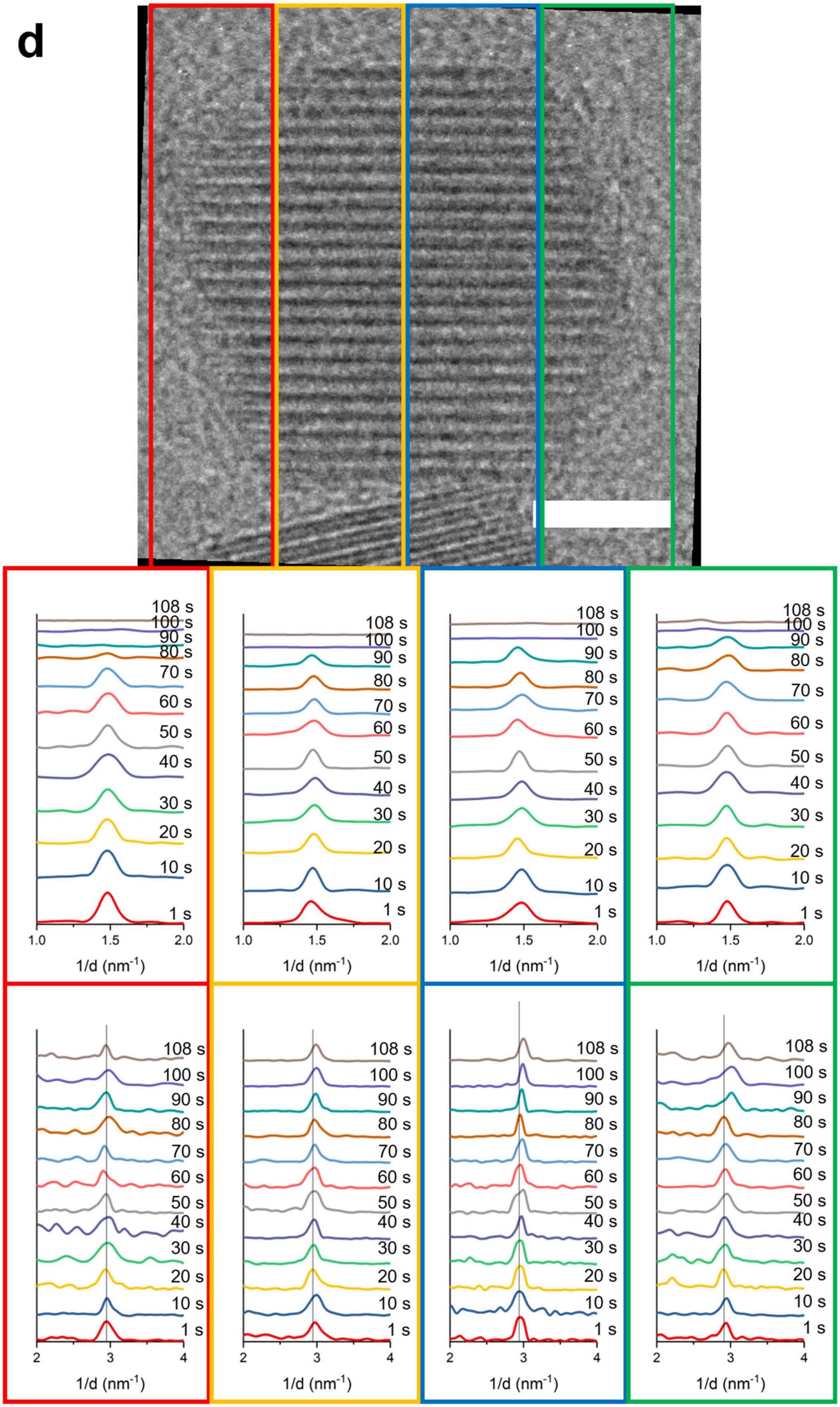

108 s
100 s
90 s
80 s
70 s
60 s
50 s
40 s
30 s
20 s
10 s
1 s
1.0
1.5
2.0
1/d (nm^{-1})
2
3
4
1/d (nm^{-1})

e

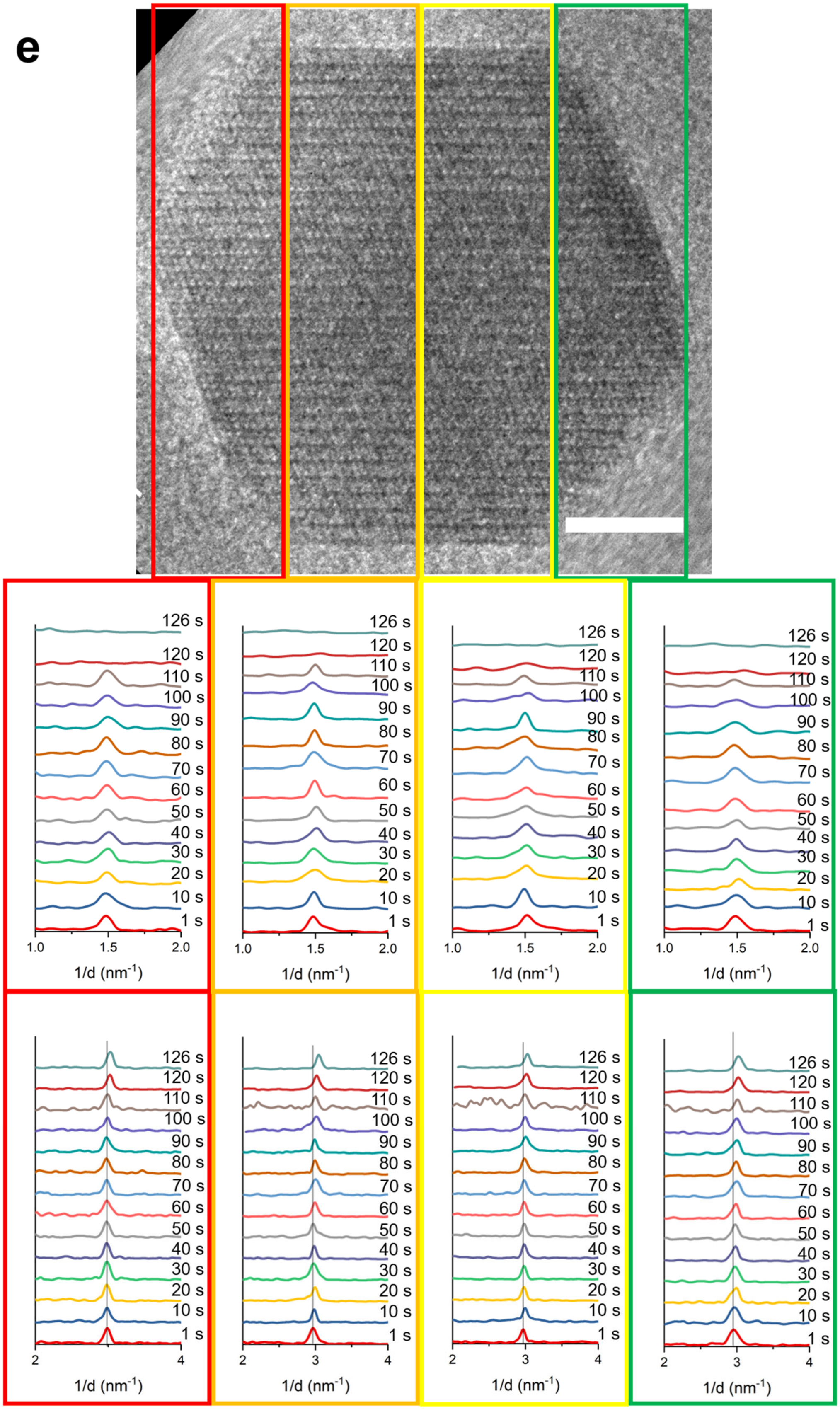

126 s
120 s
110 s
100 s
90 s
80 s
70 s
60 s
50 s
40 s
30 s
20 s
10 s
1 s
1.0
1.5
2.0
1/d (nm^{-1})
2
3
4

f

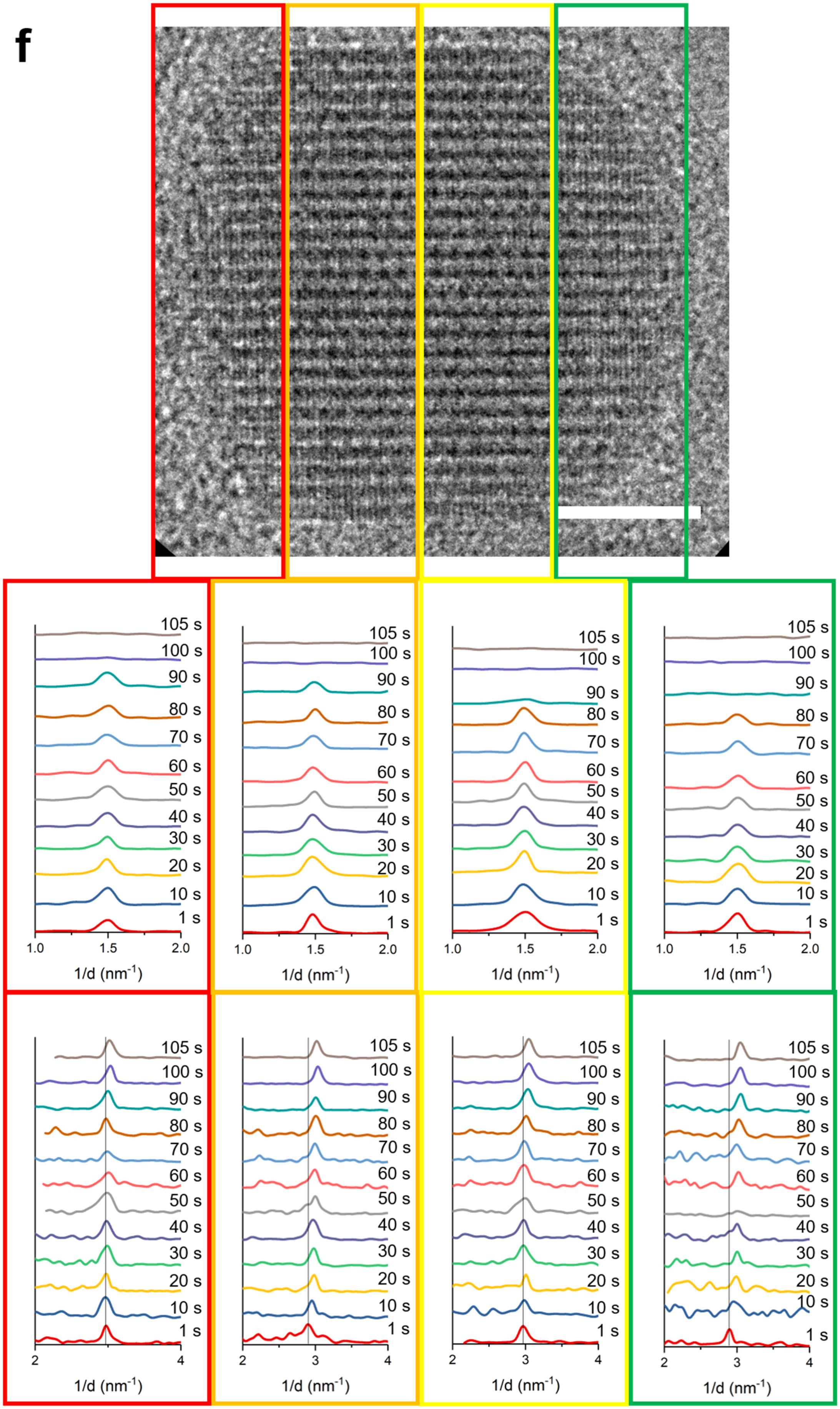
105 s
100 s
90 s
80 s
70 s
60 s
50 s
40 s
30 s
20 s
10 s
1 s
1.0
1.5
2.0
2
3
4
1/d (nm⁻¹)

g

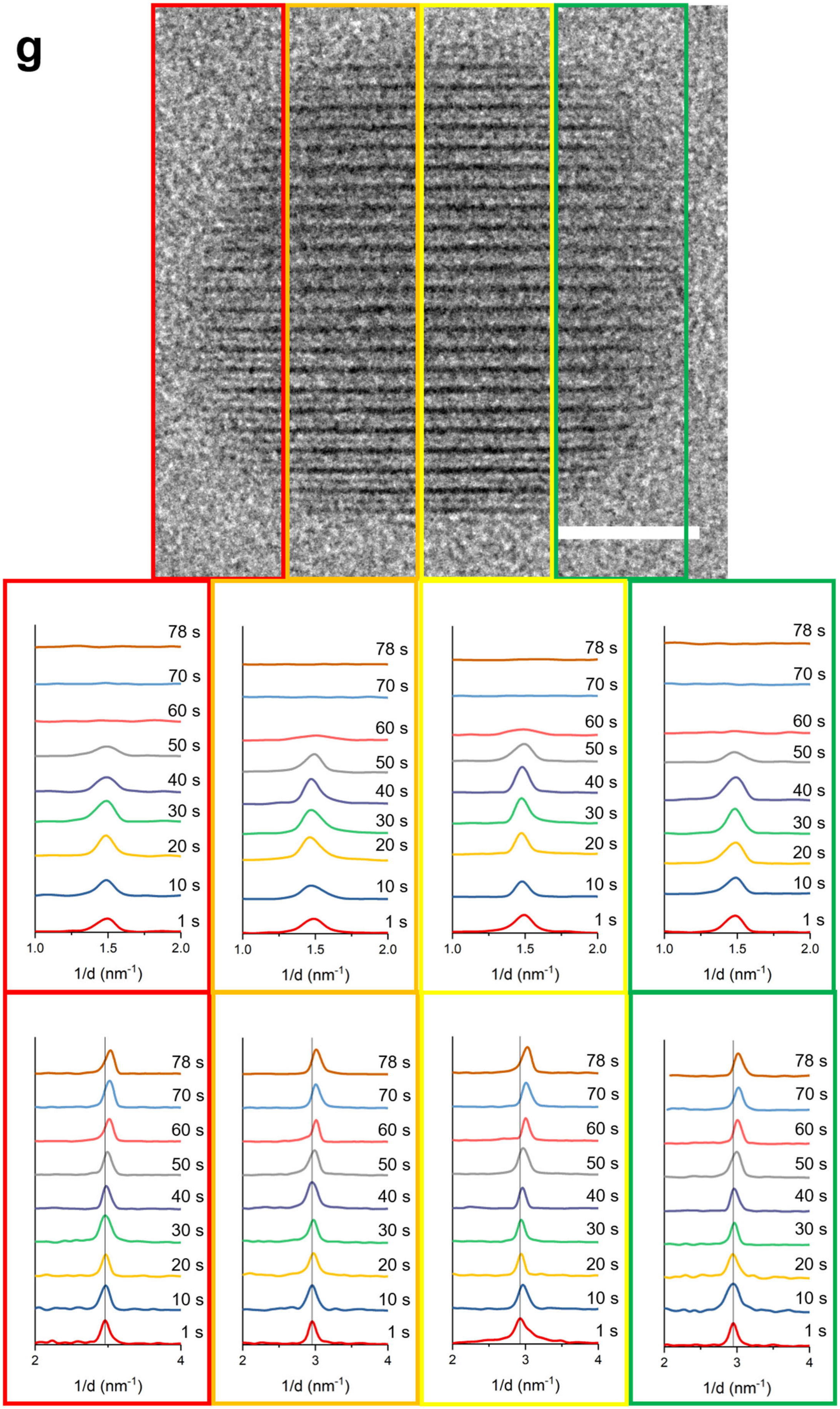

h

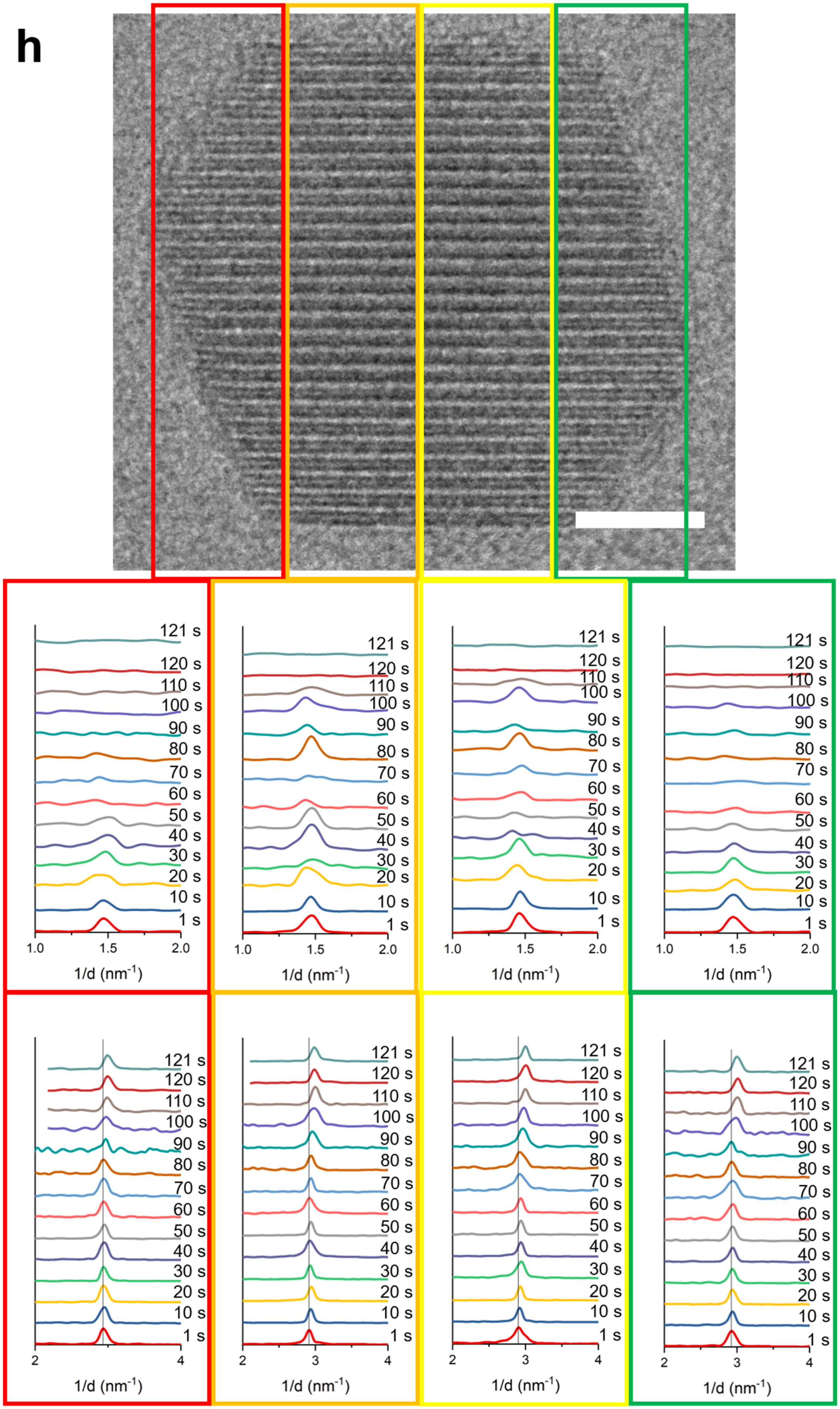
121 s
120 s
110 s
100 s
90 s
80 s
70 s
60 s
50 s
40 s
30 s
20 s
10 s
1 s
1.0
1.5
2.0
1/d (nm^{-1})
2
3
4

i

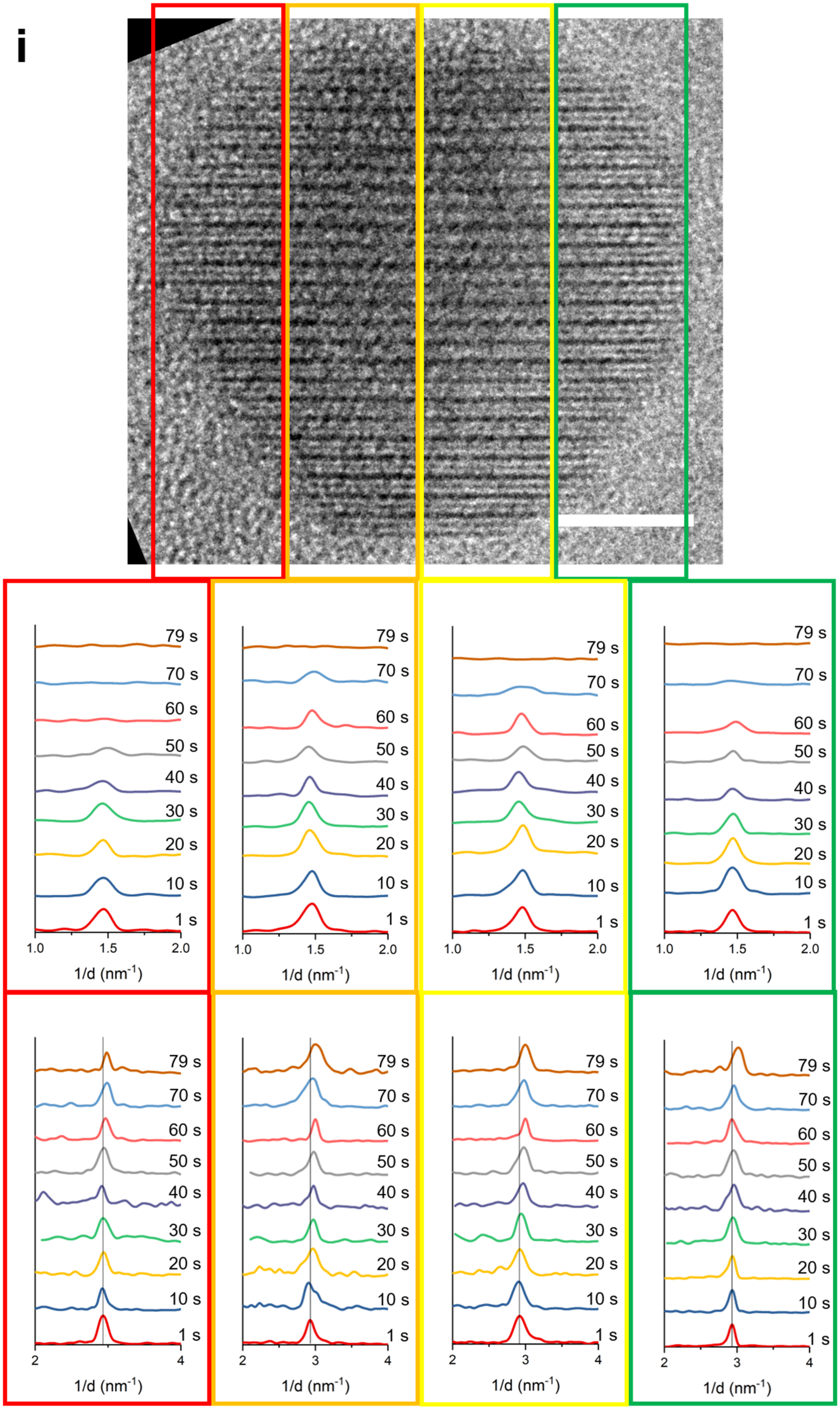

Supplementary Figure 6. To supplement the representative case shown in Fig. 3, (a–i) additional examples of PDFs as a function of time are shown for 9 additional HNPs. The top row shows the HRTEM image of the HNP oriented with the $[111]_c$ direction vertical. Scale bars are 5 nm in length for all images. Note that the HNPs shown in Supplementary Figure 6 b, c, and d correspond to the same ones shown in the top row of Fig. 1c, middle row of Fig. 1c, and bottom row of Fig. 1c, respectively. The oriented HNP lattice is sub-divided into four distinct regions (outlined by the four colored boxes). The middle row shows the PDFs in the d^{-1} = 1–2 nm^{-1} range for each of the four regions in the corresponding four columns. Every PDF was normalized by dividing by the maximum of the PDF with the highest intensity peak in that series. The bottom row shows the PDFs in the d^{-1} = 2–4 nm^{-1} range for each of the four regions in the corresponding four columns. Every PDF was separately normalized from 0 to 1. The PDFs are stacked vertically as a function of the time-point in the movie. The vertical line is aligned with the reciprocal-lattice peak maximum at the starting time-point. The PDFs show that in every case, the reciprocal-lattice peak around 3 nm^{-1}, which corresponds to the Se^{2-}-Se^{2-} interplanar spacing along $[111]_c$, shifts to smaller *d*-spacings at a time alongside the loss of the Cu-vacancy super-lattice peak around 1.5 nm^{-1}. This sequence indicates that the compression of the Se^{2-} sub-lattice accompanies the loss of Cu-vacancy ordering. However, it appears that in rare cases, e.g., left-most columns in Supplementary Figure 6a and d, there is a reversal in the Se^{2-}-Se^{2-} *d*-spacing trend at later times. These cases involve PDFs obtained from the region near the boundaries of the HNP; the overall number of lattice planes contained within the region is relatively small. So, either the *d*-spacing measured from these boundary regions is less reliable or these boundary regions exhibit potential fluctuations in the *d*-spacing. This is why it is important to determine the *d*-spacing by averaging all four regions of the HNP (as carried out for the results in Fig. 3), so that the measured *d*-spacing is reliable and representative of the lattice parameter of the entire HNP.

Supplementary References

1. Reimer, L. & Kohl, H., Transmission Electron Microscopy: Physics of Image Formation, Ch. 11 (Springer, 2008).
