# Supplementary material for: In-situ electron microscopy mapping of an order-disorder transition in a superionic conductor
Source: Nat Commun. 2019 Apr 3;10:1505. doi: 10.1038/s41467-019-09502-5 (PMC6447557; doi:10.1038/s41467-019-09502-5)
Supplement: Supplementary file 2 — Description of Additional Supplementary Files [file 41467_2019_9502_MOESM2_ESM.docx]

**Description of Additional Supplementary Files**

File Name: Supplementary Movie 1

Description: HRTEM movie showing the time-evolution of the real-space lattice structure of a Cu_2_-_x_Se HNP that is undergoing an electron-beam-induced phase transition from the VO to the SI phase. HNP shown in this video corresponds to the one presented in Fig. 1A. The movie was acquired at 1 frame s^-1^ but is played here at 10× the actual frame rate. The timestamp (s) is shown in the top right corner.

File Name: Supplementary Movie 2

Description: Movie showing the time-evolution of the reciprocal lattice of a Cu_2_-_x_Se HNP that is undergoing an electron-beam-induced phase transition from the VO to the SI phase. Upon phase transition, the reciprocal-lattice spots corresponding to the Cu-vacancy superlattice (marked by red arrows) disappear. This movie was generated in ImageJ software by FFT of each frame of the real-space Movie M1, which was followed by adjustment of brightness and contrast. The movie is played at the actual frame rate of 1 frame s^-1^. The time-stamp (s) is shown in the top right corner.
